# Physical factors that differentiate body kinematics between treadmill and overground walking

**DOI:** 10.3389/fbioe.2022.888691

**Published:** 2022-08-26

**Authors:** Mingi Jung, Seungbum Koo

**Affiliations:** Department of Mechanical Engineering, Korea Advanced Institute of Science and Technology, Daejeon, South Korea

**Keywords:** gait kinematics, treadmill walking, gait controller, reinforcement learning, machine learning

## Abstract

Treadmills are widely used in rehabilitation and gait analysis. However, previous studies have reported differences in terms of kinematics and kinetics between treadmill and overground walking due to physical and psychological factors. The aim of this study was to analyze gait differences due to only the physical factors of treadmill walking. Walking motions of a male participant were captured at 0.63, 1.05, 1.33, and 3.91 m/s. A gait controller of a virtual subject (63 kg) was trained for ground walking at each walking speed *via* a reinforcement learning method. Additionally, the gait controllers of virtual subjects with different body masses of 47, 79, and 94 kg were trained for ground walking at 1.05 m/s. The gait controllers and virtual subjects were tested for treadmill walking, and their lower-limb joint kinematics were compared with those for ground walking. Treadmill conditions of maximum allowable belt force and feedback control frequency of belt speed were set between 100 and 500 N and between 10 and 50 Hz, respectively. The lower-limb kinematics were identical between the two conditions regardless of the body mass and walking speed when the belt could provide a constant speed regardless of external perturbation in the ideal treadmill. However, kinematic differences were observed when simulation was performed on a non-ideal treadmill with a relatively low belt force and control frequency of belt speed. The root-mean-square differences of the hip, knee, and ankle flexion angles between treadmill and overground running at 3.91 m/s increased by 3.76°, 3.73°, and 4.91°, respectively, when the maximum belt force and control frequency decreased from infinity to 100 N and 10 Hz, respectively. At a maximum belt force exceeding 400 N or a control frequency exceeding 25 Hz, the root-mean-square difference of the joint kinematics was less than 3° for all body masses and walking speeds. Virtual subjects walking on non-ideal treadmills showed different joint kinematics from ground walking. The study identified physical factors that differentiate treadmill walking from overground walking, and suggested the belt forces and control frequencies of a treadmill to achieve the desired limit of kinematic differences.

## Introduction

Treadmills are widely used in exercise, gait analysis, and rehabilitation training (Colombo et al., 2000; Pohl et al., 2002; Pohl et al., 2003; Fung et al., 2006; Park et al., 2013) because they provide a convenient way to walk and run in a relatively narrow indoor space. Meanwhile, a number of studies reported that walking on a treadmill is different from overground walking (Elliott and Blanksby, 1976; Pearce et al., 1983; Dingwell et al., 2001; Nymark et al., 2005; Dasilva et al., 2011). Previous studies showed that body kinematics, such as hip and knee flexion angles, differed between treadmill and overground walking (Alton et al., 1998). Kinetic parameters, such as the sagittal plane joint moment during walking, also differed between the two walking conditions (Lee and Hidler, 2008).

The differences between treadmill and overground walking have been hypothesized to be caused by visual information, air resistance, physical factors such as irregular treadmill belt speed, and psychological factors (Gates et al., 2012; Goodworth et al., 2015; Léger and Mercier, 1984; Malatesta et al., 2017; Pugh, 1970; Tielke et al., 2019; Traballesi et al., 2008; Willwacher et al., 2021). The preferred walking speed on treadmill was lower than that on ground (Malatesta et al., 2017), which was associated with psychological causes (Traballesi et al., 2008). Oxygen intake during running in the overground condition was higher than that in the treadmill condition because of air resistance (Pugh, 1970; Léger and Mercier, 1984), meanwhile treadmill walking demanded a higher metabolic cost of transport than the overground walking possibly due to an anxiety of walking in a constrained space (Martin and Li, 2017). Additionally, physical causes affected the gait differences between treadmill and overground conditions. The belt speed fluctuated depending on the gait phase, walking speed, and body mass (Tielke et al., 2019; Willwacher et al., 2021), which should be due to the absence of feedback speed control mechanisms such as a servo system (Wu et al., 2021).

However, it appeared that all these factors contributed to the differences between overground and treadmill walking. The effects of individual human and treadmill factors are not well understood. Immersive projection technologies have been used along with treadmills to improve walking conditions by providing visual information similar to that of ground walking (Winter et al., 2021). Meanwhile, the isolated effects of physical factors such as treadmill power, belt speed control frequency, body mass, and walking speed on body kinematics during treadmill walking are yet to be elucidated.

Recent developments in machine learning have enabled the motion controller of a multibody dynamics system in a dynamic simulator to be trained to perform tasks such as walking and running (Duan et al., 2016). Using imitation learning methods, a motion controller can be trained to track measured motion data, such as a specific person’s gait motion (Peng et al., 2018; Yuan and Kitani, 2020; Lee et al., 2019).

The purpose of this study was to investigate the contributions of purely physical factors, such as the subject’s body mass and walking speed, as well as the treadmill belt force and the belt speed control frequency for its feedback control, on the kinematic differences between treadmill and overground walking. Treadmill belt power, which is belt force times belt speed, provides a measure that is an analog to treadmill motor power. We used an imitation learning method to learn gait controllers that mimicked the gait motions of a subject, and then used these gait controllers to perform forward dynamic gait simulation in the treadmill and overground conditions to determine the effects of physical factors on body kinematic differences. In the dynamic simulator, air resistance, and psychological causes such as fear do not exist; therefore, only the effects of physical factors were investigated.

## Materials and methods

### Gait data acquisition and processing

A healthy male subject (age, 20 years; body mass, 63 kg; height, 173 cm) without a history of knee and ankle injury participated in this study after institutional review board approval and informed consent were obtained. A full-body plug-in-gait marker set was used to obtain motion capture data during walking and running using 13 motion-capture cameras. Motion data of the subject during walking on a flat ground surface at low (0.63 m/s), natural (1.05 m/s), and high (1.33 m/s) speeds and running (3.91 m/s) were captured. The kinematics of the joints or reference motions were calculated using the motion capture data *via* the inverse kinematics module in OpenSim v4.2 (Delp et al., 2007).

### Human models and gait controllers

**FIGURE 1:**
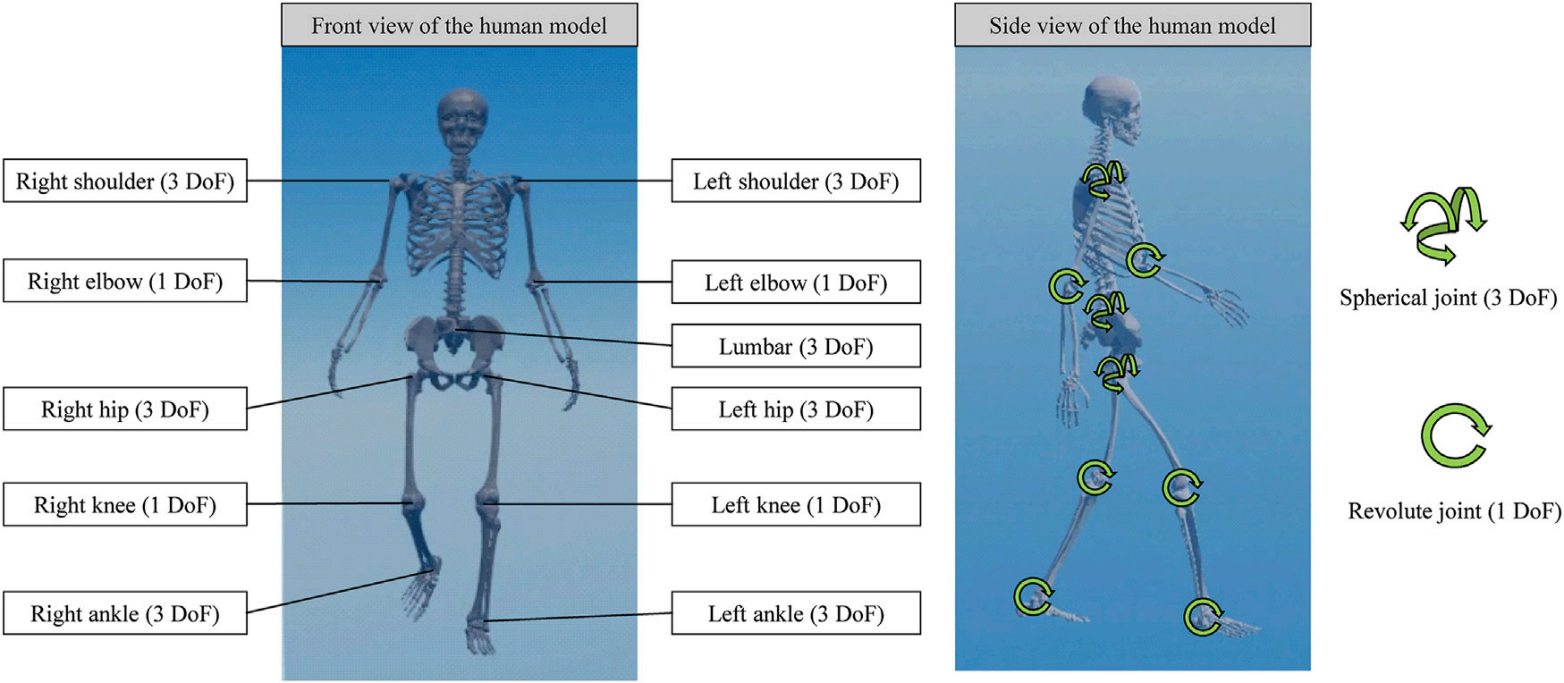
Three-dimensional human skeletal model and degree-of-freedom of joints.

Four human skeletal models were created using anatomical and geometrical information from a previous study (Rajagopal et al., 2016). The human models exhibited identical structures; however, the total body masses differed as detailed below. The human model (Figure 1) comprised ideal actuatable joints with a total of 25 degrees of freedoms (DoFs), including two shoulders (each with three DoFs), two elbows (each with one DoF), a lumbar (three DoFs), two hips (each with three DoFs), two knees (each with one DoF), and two ankles (each with three DoFs).

The dynamics simulation environment was created using RaiSim (Hwangbo et al., 2018), which is a multipurpose dynamics engine. For the forward dynamics simulation of the human skeletal model, 25 actuatable joints should be actuated harmoniously and sequentially using a controller. A neural network with two hidden layers comprising 256 × 256 nodes was used to create a gait controller. The gait controllers of the four human skeletal models were trained using deep reinforcement learning, as follows:

First, we prepared human models and reference motions. Four types of human models were prepared: models with 75%, 100%, 125%, and 150% of the subject’s original body mass of 63 kg. Their body masses were 47, 63, 79, and 95 kg, respectively. The body mass indices of the four models ranged from 15.7 (moderate thinness) to 31.6 (obese). Four reference motions were prepared: low-, natural-, high-speed walking motions, and a natural-speed running motion. Their speeds were 0.63, 1.05, 1.33, and 3.91 m/s, respectively.

Second, a reward function was established for the reinforcement learning. In reinforcement learning, the reward function serves as a loss function, and the gait controllers are trained to increase the sum of the rewards for a certain period. The reward function was a weighted sum of pose reward $\left(r^{p}\right)$ , velocity reward $\left(r^{v}\right)$ , end-effector reward $\left(r^{e}\right)$ , and center-of-mass reward $\left(r^{c}\right)$ .

$$\begin{array}{c} r=\;w^{p}r^{p}+\;w^{v}r^{v}+\;w^{e}r^{e}+\;w^{c}r^{c}\\ w^{p}=0.65,\;w^{v}=0.1,\;w^{e}=0.15,\;w^{c}=0.1 \end{array}$$

We used the weights of rewards in a previous study (Peng et al., 2018). The detailed expressions and meanings of each reward are as follows:

$$r^{p}=\exp\left[-2\left(\sum_{j}{\left\|{\hat{\theta }}^{j}-\theta^{j}\right\|}^{2}\right)\right]\;.$$

$$r^{v}=\exp\left[-0.04\left({\sum_{j}\left\|{\hat{\dot{\theta }}}^{j}-{\dot{\theta }}^{j}\right\|}^{2}\right)\right]\;.$$

The pose reward $r^{p}$ and velocity reward $r^{v}$ encourage the actuatable joints of the human model to match the joint angles and velocities of the reference motion, respectively. Here, θ *,* $\dot{\theta }$ *,* $\hat{\theta }$ , and $\hat{\dot{\theta }}$ denote the joint angles and velocities of the human models and reference motions, respectively, where *j* denotes the joint index of the human model. The pose and velocity rewards were calculated by comparing the joint angles and velocities with the reference motions at every time step. At the three-DoF joints, the differences in the angles and velocities were computed using the quaternion differences.

$$r^{e}=\exp\left[-40\left({\sum_{e}\left\|{\hat{p}}^{e}-p^{e}\right\|}^{2}\right)\right]\;.$$

The end-effector reward $r^{e}$ encourages the end-effectors of the human model to monitor the reference motion. Here, $p^{e}$ and ${\hat{p}}^{e}$ denote the positions of the end-effector of the human model and reference motion, respectively, representing the three-dimensional position relative to the pelvis position. The end-effectors included the left hand, right hand, left foot, and right foot.

$$r^{c}=\exp\left[-30\left({\left\|{\hat{p}}^{c}-p^{c}\right\|}^{2}\right)\right]\;.$$

The center-of-mass reward $r^{c}$ was computed based on the difference between the human model and reference motion. Here, $p^{c}$ and ${\hat{p}}^{c}$ denote the center-of-mass positions of the human model and reference motion, respectively, representing the three-dimensional position relative to the pelvis position.

Third, the observations and actions were the input and output vectors, respectively, of the gait-controller networks. When the human model was simulated in a dynamic environment, kinematics, and kinetics information could be extracted. The observation was a vector of 86 elements. The components (and their number of elements) of the observations were pelvis height (1), pelvis orientation (3), pelvis linear velocity (3), pelvis angular velocity (3), angles of all movable joint (25), angular velocities of all movable joint (25), gait phase (1), and terrain height at 5 by 5 grids around the model (25). The actions of the gait controller network were the target positions of all movable joints (25). The action vector was input to a PD (proportional derivative) controller to calculate torques of the movable joints (Peng and Van, 2017).

**FIGURE 2:**
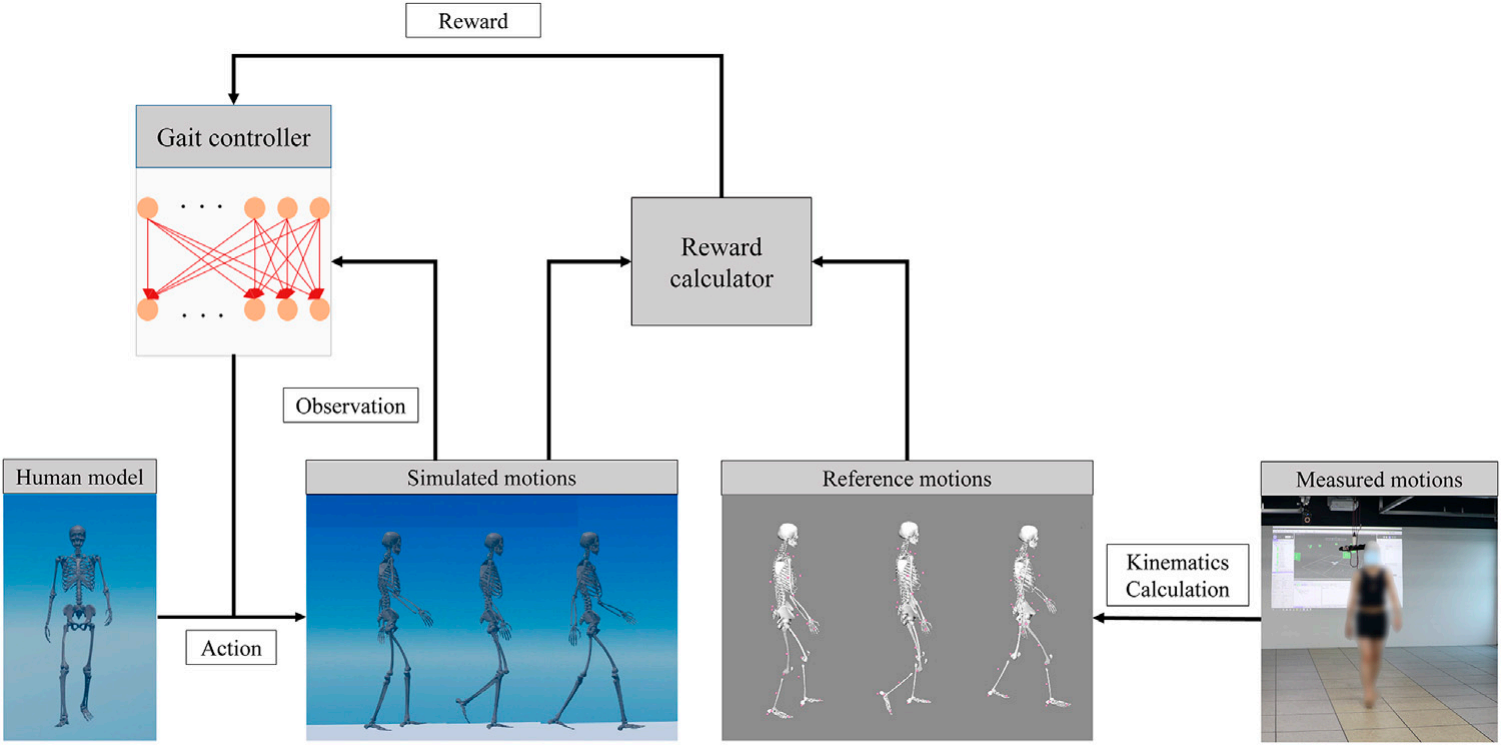
Training process for gait controllers using reinforcement learning.

Finally, the parameters of the gait controller network were updated using an optimization method. We updated the gait controllers using the proximal policy optimization (PPO) algorithm (Schulman et al., 2017). The learning process is illustrated in Figure 2. The human model walked in a dynamic environment by action, and the extracted observations became the input of the gait controller network. The network parameters were updated using the PPO algorithm based on the reward calculated by comparing the state of the human model with that of the reference motion.

### Treadmill in dynamic simulation

**FIGURE 3:**
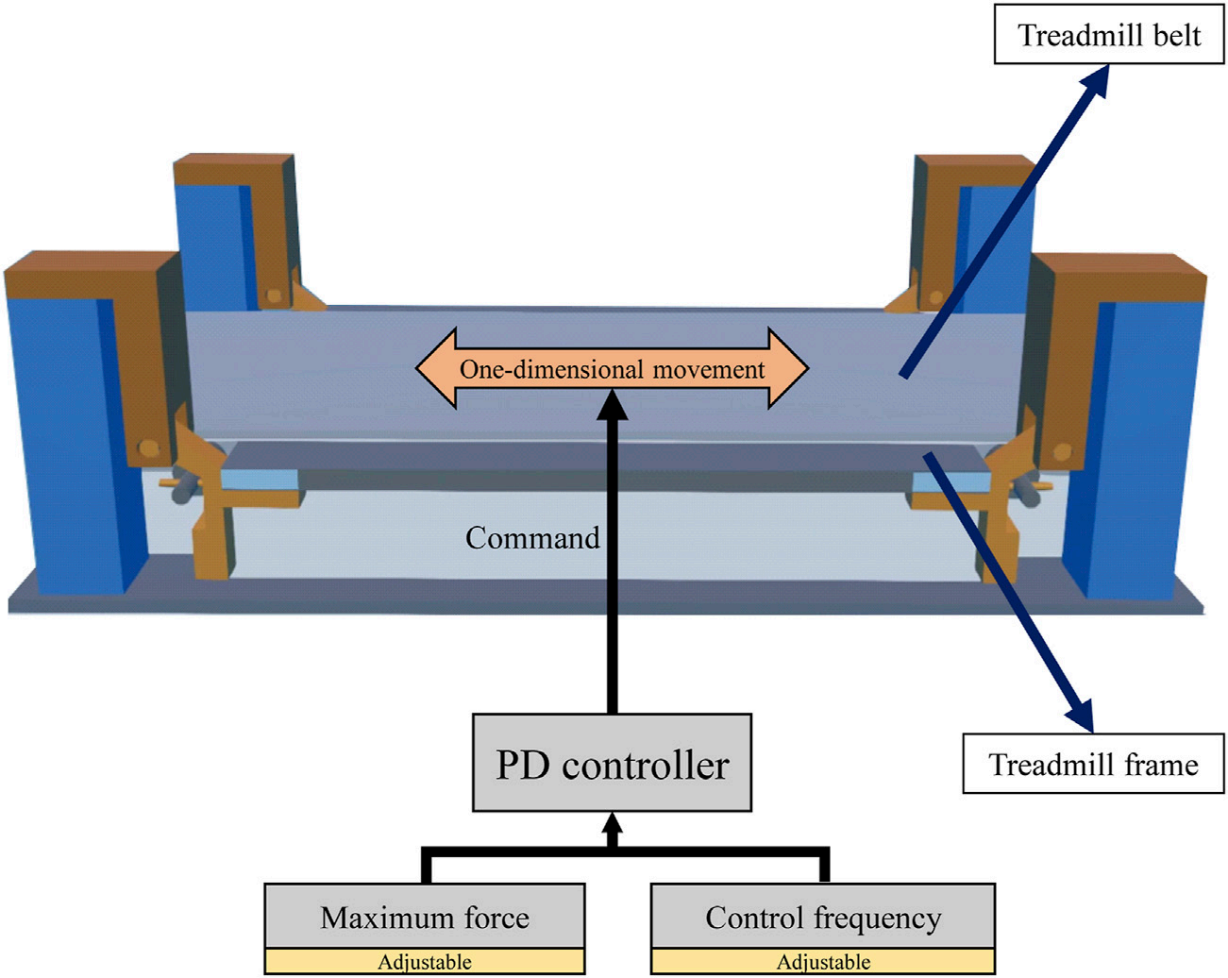
Treadmill model used for simulation.

To compare overground and treadmill walking, we created a virtual treadmill (Figure 3) comprising a fixed body frame and moving belt. The treadmill parameters, such as inertia, mass, and coefficient of sliding friction, were determined based on a previous study (Hollerbach et al., 2000). The fixed body frame and moving belt were connected by a prismatic joint. A PD controller was used to control the belt at a designated speed. Linear forces from the belt PD controller were applied to the belt to generate a linear motion. To maintain the desired belt speed, the linear force exerting on the belt was adjusted using the PD controller which includes feedback loops (Franklin et al., 2015). The maximum belt force and frequency of the belt PD controller were set as the experimental parameters to simulate walking under different treadmill conditions. The P gain and D gain of the belt PD controller at each maximum belt force and control frequency were adjusted appropriately before the gait controllers were tested (Ellis, 2012).

### Forward dynamics simulation

Gait controllers for the standard model with body mass of 63 kg were trained for the four walking and running speeds (0.63, 1.05, 1.33, and 3.91 m/s). Additionally, for the models with the other body masses (47, 79, and 94 kg), gait controllers were trained only at the normal walking speed (1.05 m/s). Thus, total seven gait controllers were trained.

**FIGURE 4:**
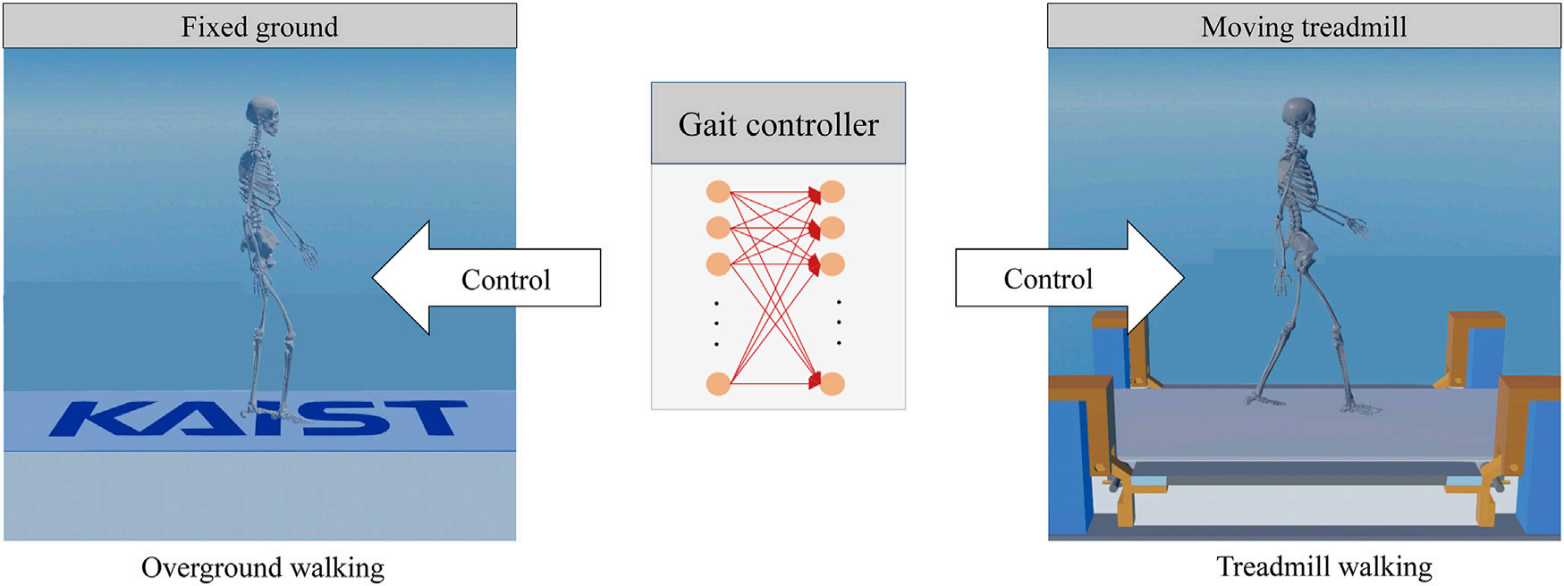
Forward dynamics simulations in overground and treadmill walking conditions using the same gait controller that was trained for overground walking.

Each gait controller was tested under overground and treadmill walking conditions (Figure 4). For the treadmill walking conditions, firstly, five different control frequencies (10, 13, 17, 25, and 50 Hz) were used for feedback control of belt speed with a maximum belt force of 300 N. The five control frequencies match the control of be belt speed at every 100, 80, 60, 40, and 20 milliseconds, respectively, to mimic a servo motor. Secondly, for a fixed control frequency of 17 Hz, maximum belt forces of 100, 200, 300, 400, and 500 N were used as the treadmill walking conditions. Thirdly, an ideal treadmill that can provide the target belt speed regardless of external perturbation was used. In the dynamics simulation the ideal treadmill was modeled as a kinematic body, in other words, a body with an infinitely large mass at constant speed. We can also think that the ideal treadmill has a motor that can provide infinitely large force (or torque) so that it can keep the treadmill run at a target speed regardless of external perturbation force. Thus, an overground condition and ten different treadmill conditions were used to test the gait controllers.

### Analysis

The effects of four physical factors, i.e., the subject’s body mass, subject’s walking speed, maximum belt force, and control frequency of the treadmill, on the kinematic differences between treadmill and overground walking were investigated. Only one factor was changed, whereas the other factors were fixed in each repeated forward dynamic simulation. The hip, knee, and ankle flexion angles measured during treadmill walking in each case were compared with those measured during the overground walking simulations using the same gait controller. The maximum and root-mean-square (RMS) differences in the hip, knee, and ankle flexion angles were measured to quantify the differences in the human body kinematics between overground and treadmill walking.

## Results

**FIGURE 5:**
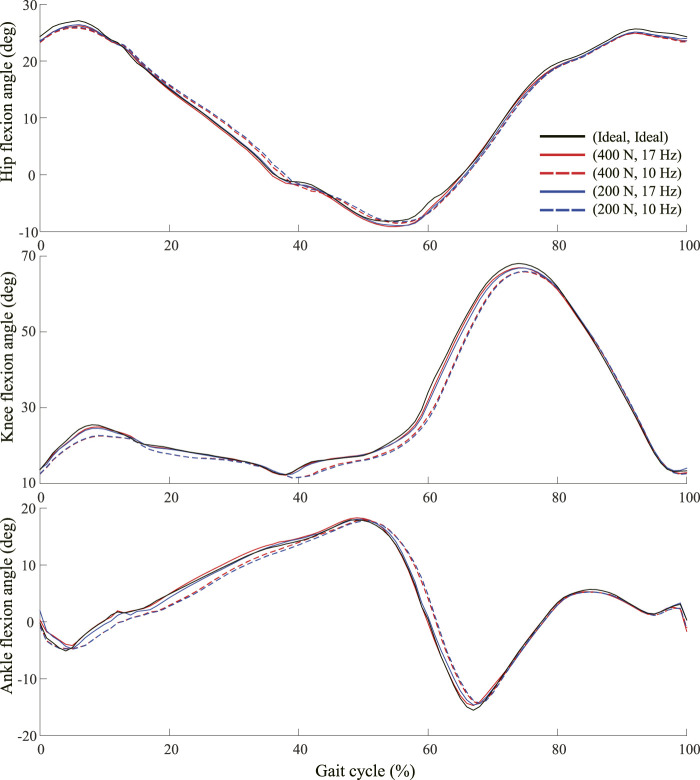
Hip, knee, and ankle flexion angles based on percent gait cycle at five different treadmill conditions based on 73 kg body mass and 1.05 m/s walking speed.

The seven gait controllers trained with the subject kinematics data could make the human model walk and run with the joint kinematics close to the subject’s kinematics. The sum of rewards of the training reward function were higher than 0.97 out of 1.0. The RMS differences of joint kinematics were lower than 3 degrees for all lower-limb joints. The joint kinematics were identical during overground and walking on the ideal treadmill which provided a constant belt speed regardless of external perturbation forces. Meanwhile, the finite and relative low belt force and control frequency of the non-ideal treadmills affected the joint kinematics during walking and running (Figure 5; Table 1). The maximum difference in the knee flexion angle was 2.07°, with a maximum belt force of 400 N and a control frequency of 17 Hz. The maximum difference in the knee flexion angle increased to 3.02° and 7.35° when only the belt force and control frequency were reduced to 200 N and 10 Hz, respectively. In general, the kinematic changes in the knee were larger than those in the hip and ankle owing to the finite and relatively low belt force and control frequency. The maximum flexion angle differences in the hip, knee, and ankle were 1.89°, 8.12°, and 5.08°, respectively, at a maximum belt force of 200 N and a control frequency of 10 Hz.

**TABLE 1:** Maximum and RMS differences of hip, knee, and ankle flexion angles between treadmill and overground walking at four different treadmill conditions.

Body mass: 63 kg		Treadmill specifications (maximum belt force, control frequency)			
Walking speed: 1.05 m/s		(400 N, 17 Hz)	(400 N, 10 Hz)	(200 N, 17 Hz)	(200 N, 10 Hz)
Maximum difference (°)	Hip	1.28	1.91	1.61	1.89
	Knee	2.07	7.35	3.02	8.12
	Ankle	1.98	4.36	2.14	5.08
RMS difference (°)	Hip	0.58	0.90	0.58	0.96
	Knee	0.65	2.50	1.00	2.70
	Ankle	0.48	1.40	0.58	1.61

**FIGURE 6:**
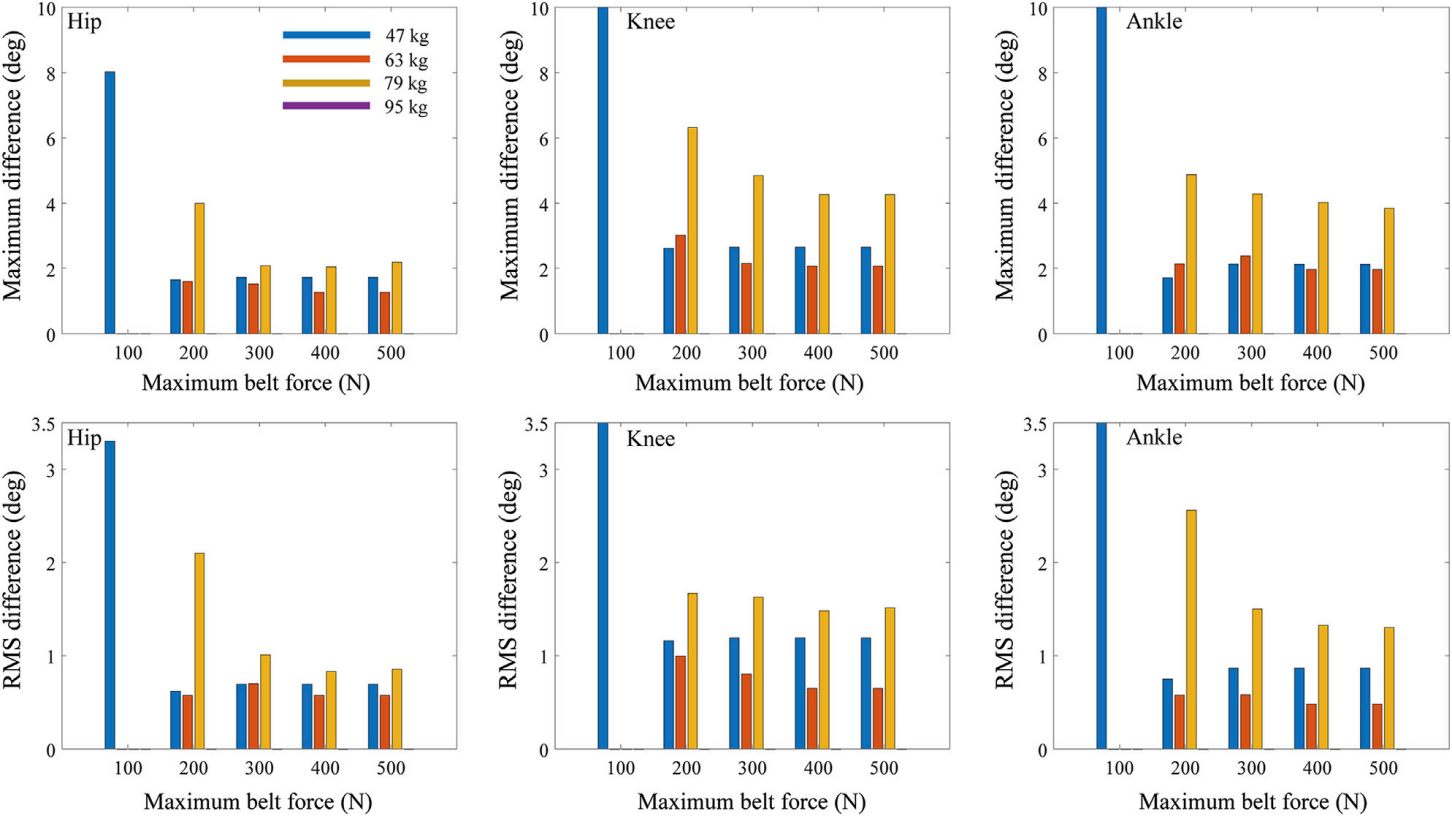
Maximum and RMS differences of joint angles between treadmill and overground walking based on maximum belt force of treadmill and body mass.

**FIGURE 7:**
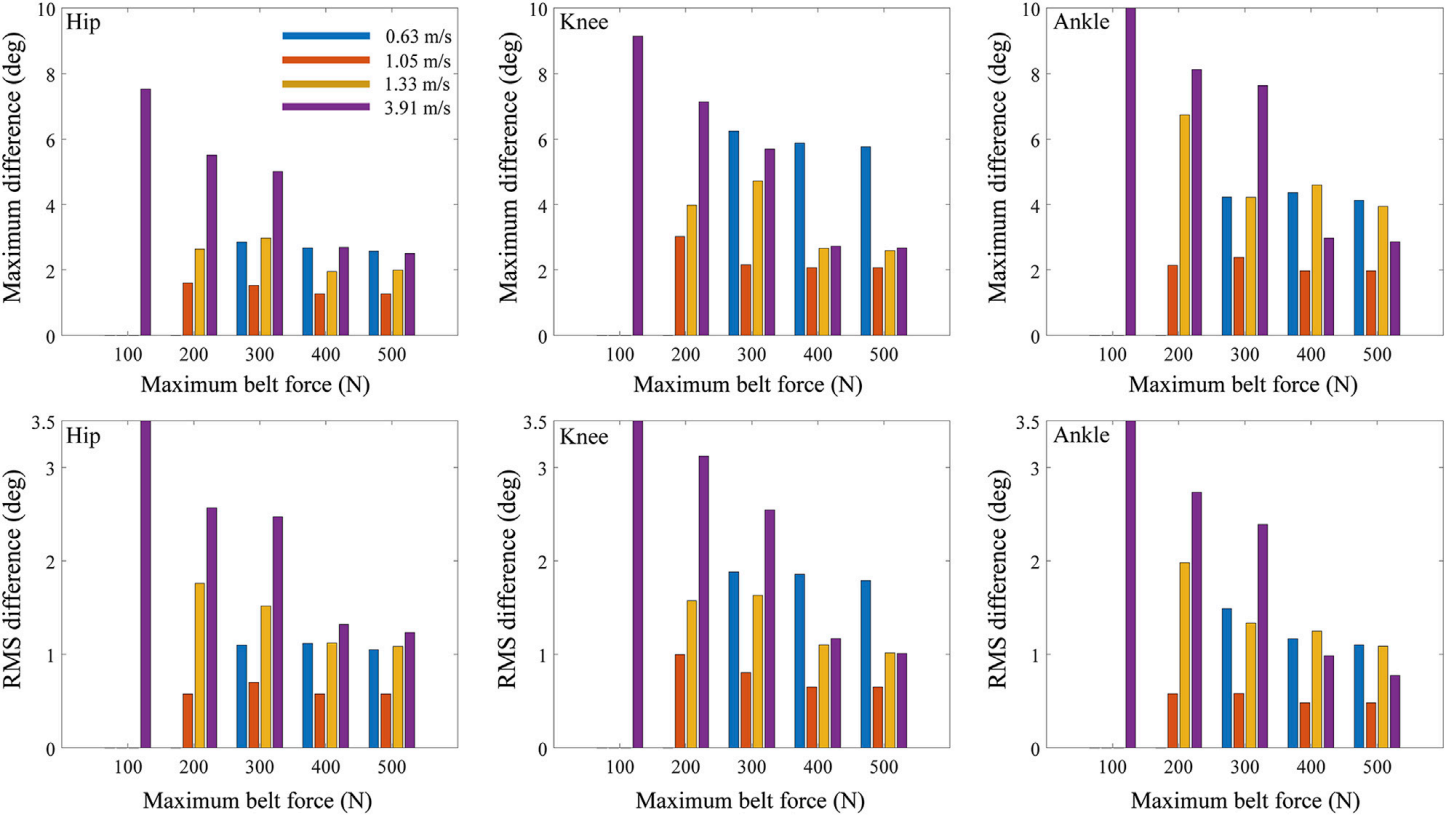
Maximum and RMS differences of joint angles between treadmill and overground walking based on maximum belt force of treadmill and walking speed.

The maximum and RMS differences in joint angle between treadmill and overground walking increased as the maximum belt force of the treadmill decreased from 500 to 100 N for all tested body masses (Figure 6) and walking speeds (Figure 7) when the control frequency was fixed at 17 Hz. When the maximum belt force of the treadmill was the lowest (100 N), the virtual subjects fell. In the case of light subjects weighing 47 and 63 kg, the decrease in RMS differences based on the maximum force of the treadmill was trivial (Figure 6, bottom).

**FIGURE 8:**
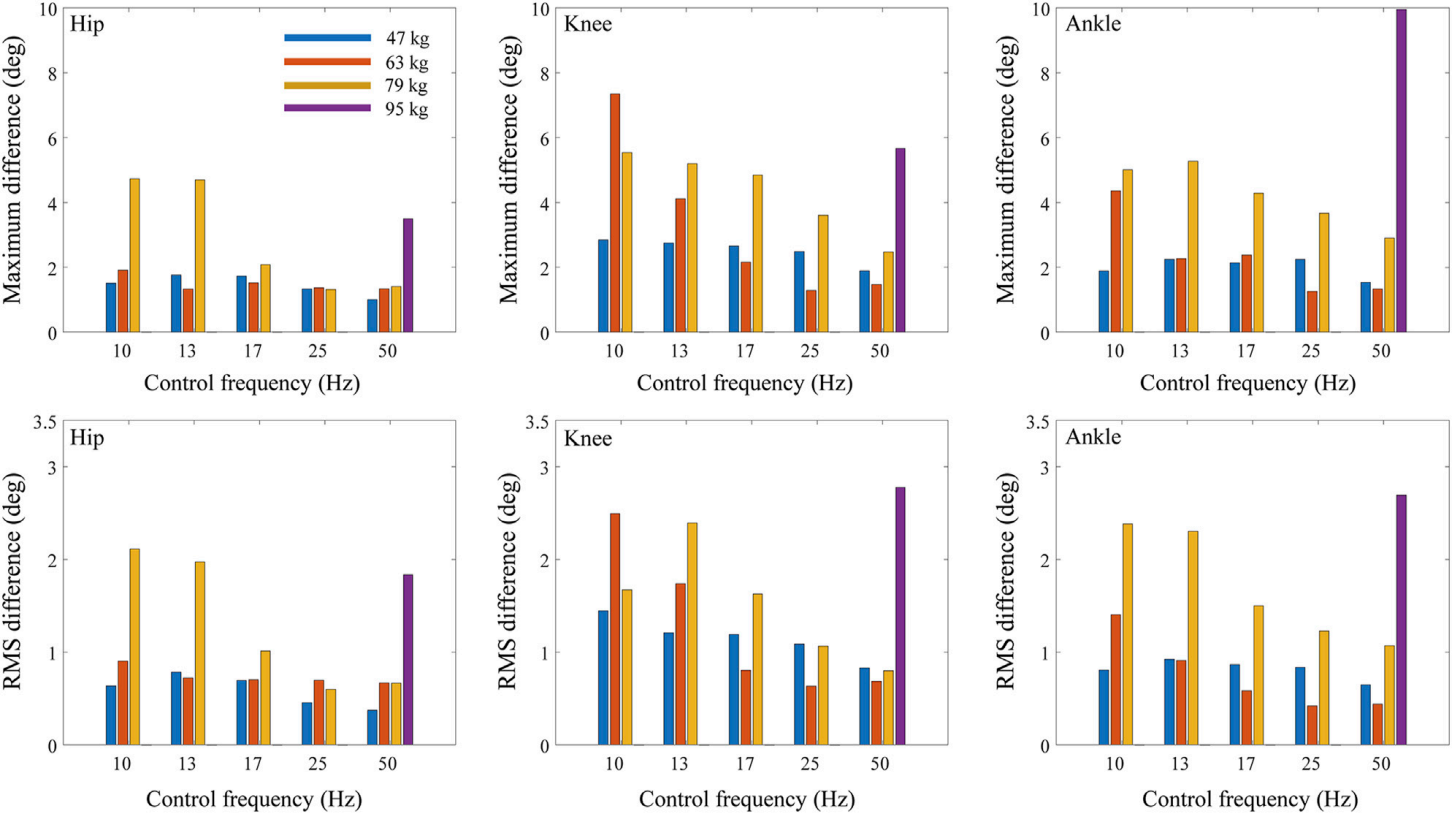
Maximum and RMS differences of joint angles between treadmill and overground walking based on control frequency of treadmill and body mass.

**FIGURE 9:**
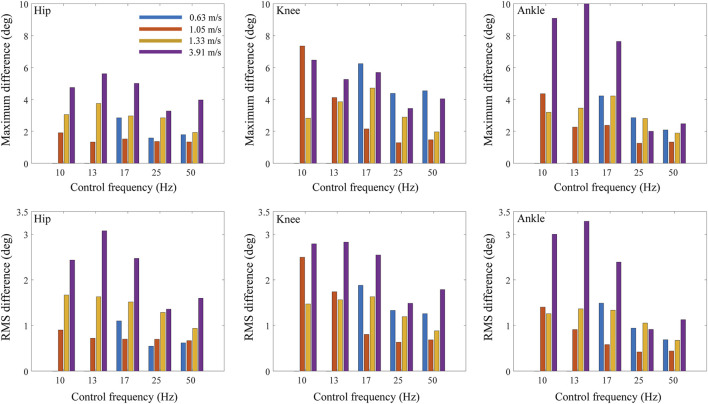
Maximum and RMS differences of joint angles between treadmill and overground walking based on control frequency of treadmill and walking speed.

When the maximum belt force was fixed at 300 N and the control frequency was decreased from 50 to 10 Hz, the maximum and RMS differences in the joint kinematics between treadmill and overground walking increased in general for all the tested body masses (Figure 8) and walking speeds (Figure 9). However, in the case of the subject with the lowest body mass (47 kg), the change in RMS differences based on the control frequency of the treadmill was less than 0.62°. Meanwhile, the hip flexion angle was affected less by the control frequency as compared with the knee and angle flexion angles.

The maximum and RMS differences in the joint angles increased in general as the body mass increased (Figures 6, 8; Table 2). The RMS differences of the 47 kg subject in the hip, knee, and ankle flexion angles were 0.78, 1.29, and 0.73°, respectively, with a maximum belt force of 200 N, control frequency of 13 Hz, and walking speed of 1.05 m/s. However, the RMS differences in the hip, knee, and ankle flexion angles of the 79 kg subjects were 2.55°, 2.63°, and 2.47°, respectively. When the control frequency exceeded 25 Hz or the maximum belt force exceeded 400 N, the RMS differences changed slightly depending on the body mass. The difference in hip kinematics was affected less by the body mass than those of the knee and ankle. A human model weighing 95 kg fell during the walking simulations, except for the treadmill condition with the highest control frequency of 50 Hz. The maximum and RMS differences in the hip, knee, and ankle flexion angles showed similar trends.

**TABLE 2:** Maximum and RMS differences of joint angles between treadmill and overground walking at four different body masses.

Maximum belt force: 200 N		Body mass			
Control frequency: 13 Hz		47 kg	63 kg	79 kg	95 kg
Walking speed: 1.05 m/s					
Maximum difference (°)	Hip	2.07	1.67	4.36	Fell
	Knee	2.57	5.62	7.00	Fell
	Ankle	1.55	3.14	6.51	Fell
RMS difference (°)	Hip	0.78	0.77	2.55	Fell
	Knee	1.29	2.01	2.63	Fell
	Ankle	0.73	1.13	2.47	Fell

The maximum and RMS differences increased with the walking speed (Figures 7, 9; Table 3). The RMS differences with a walking speed of 1.05 m/s in the hip, knee, and ankle flexion angles were 0.77°, 2.01°, and 1.13°, respectively, at a maximum belt force of 200 N, control frequency of 13 Hz, and body mass of 63 kg. However, the RMS differences at a walking speed of 3.91 m/s in the hip, knee, and ankle flexion angles were 3.26°, 3.10°, and 4.10°, respectively. In the walking simulations at a walking speed of 0.63 m/s, falling occurred when the maximum belt force was less than 200 N or when the control frequency was less than 13 Hz. When the control frequency exceeded 25 Hz or the maximum belt force exceeded 400 N, the RMS differences changed slightly depending on the walking speed.

**TABLE 3:** Maximum and RMS differences of joint angles between treadmill and overground walking at four different walking speeds.

Maximum belt force: 200 N		Walking speed			
Control frequency: 13 Hz		0.63 m/s	1.05 m/s	1.33 m/s	3.91 m/s
Body mass: 63 kg					
Maximum difference (°)	Hip	Fell	1.67	4.66	6.67
	Knee	Fell	5.62	4.39	6.22
	Ankle	Fell	3.14	5.44	14.00
RMS difference (°)	Hip	Fell	0.77	2.23	3.26
	Knee	Fell	2.01	2.03	3.10
	Ankle	Fell	1.13	1.78	4.10

## Discussion

This study aimed to investigate the contributions of a subject’s body mass and walking speed as purely physical factors, together with the effects of equipment-related factors such as the treadmill belt force and the belt speed control frequency, on the kinematic differences between treadmill and overground walking. Using a simulation-based approach allowed us to ensure that the body kinematics were identical between the overground walking conditions and those while walking on an ideal treadmill at all treadmill speeds for all seven gait controllers. The ideal treadmill was designed to provide the constant target belt speed regardless of external forces. The frame moving with the belt is considered an inertial frame, so that the dynamics on the ground and the treadmill are theoretically and physically identical (Van, 1980; Chabay and Sherwood, 2006). This means that the dynamics of the human model with a gait controller is the same on the ground and treadmill, resulting in identical body kinematics.

A non-ideal treadmill with a finite and relatively low belt force and belt speed control frequency cannot maintain a constant target belt speed against foot braking force while walking, which inversely affects the joint kinematics and body balance. Differences between overground walking and treadmill walking have been investigated extensively; however, contradictory results have previously been reported in the literature. For example, Lee and Hidler reported that the leg kinematics between overground and treadmill walking were similar (Lee and Hidler, 2008), whereas Alton et al. (1998) reported that the hip flexion angle for females and the knee flexion angle for males were different in both conditions. These results were likely the result of a failure in controlling all factors that can cause a difference between the two conditions, such as physical factors, psychological factors, air resistance, and visual information. In the current study, except for four physical factors, the other factors were fixed in both conditions to avoid affecting gait, and the isolated effect of each physical factor was investigated.

In actual experiments, it is impossible to change only the body mass while fixing all other factors. The subject should be changed such that a different body mass can be investigated; however, because of the uniqueness of gait kinematics, the gait motion changes while other factors are changed (Park et al., 2021). However, we trained the four gait controllers with each body mass (47, 63, 79, and 95 kg) using identical geometrical models and reference motions. Therefore, the gait motions were created under overground and treadmill conditions with different body masses only, and the analysis based on body mass was performed successfully.

**FIGURE 10:**
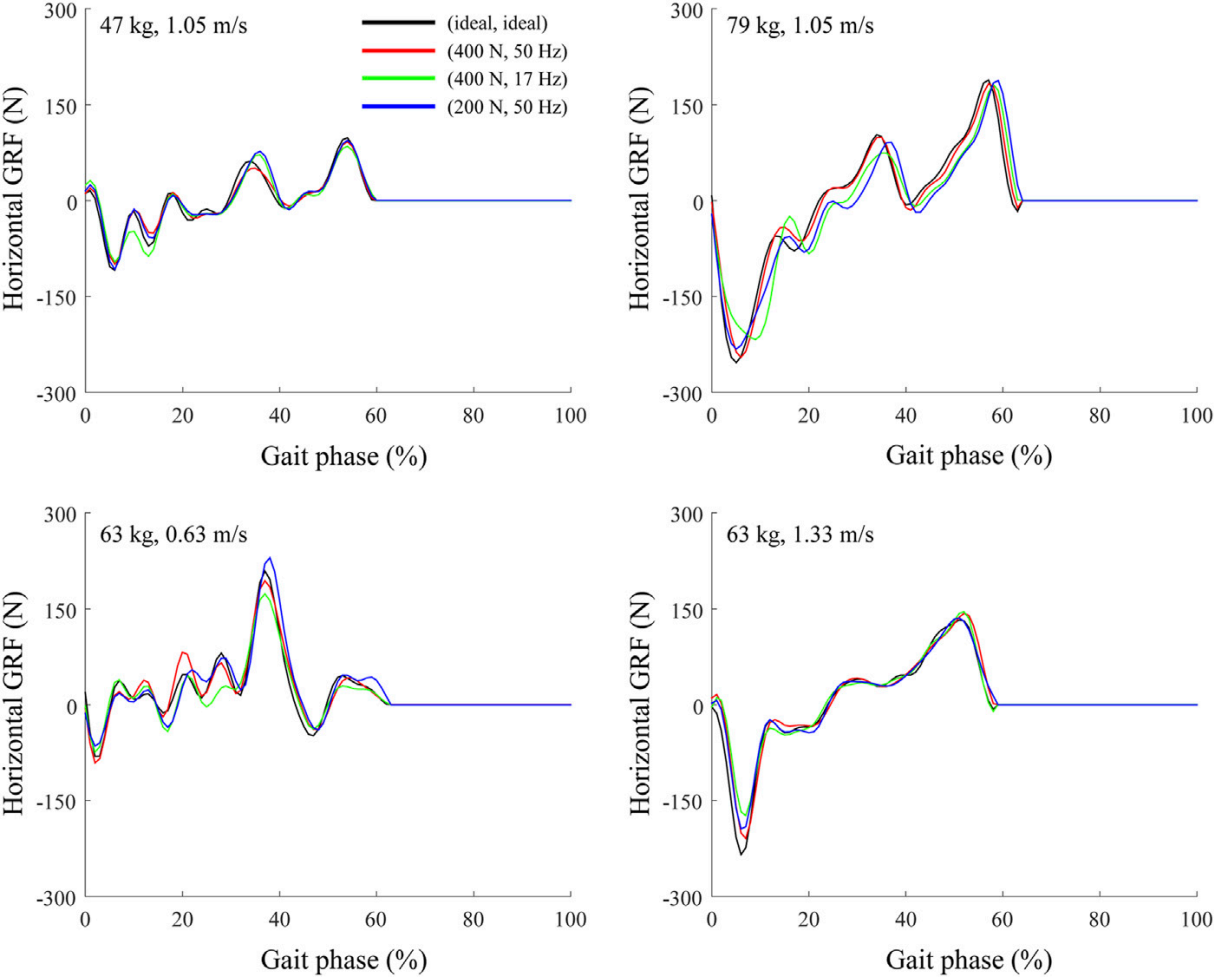
Horizontal GRF at right foot during treadmill and overground walking at four different treadmill conditions.

A human model with a large body mass fell over while walking on a treadmill with a low maximum belt force and control frequency. To analyze this phenomenon, the horizontal ground reaction force (GRF) was derived from the analysis based on the body mass and treadmill conditions (Figure 10). As the body mass increased, the difference in the horizontal GRF between the ideal and non-ideal treadmill conditions increased. On the non-ideal treadmill, the horizontal GRF appeared to be affected by a phase shift with the extent of the delay depending on the gait phase, which is consistent with the results of a previous study using force plates (Bundle et al., 2015). Because the gait controller was composed of a neural network, its robustness depended significantly on the learning method and environment (Perrusquia and Yu, 2021). Because each gait controller was trained under the overground condition, the human model fell over when the GRF changed significantly on the treadmill as compared with overground walking.

The human model fell over at the lowest walking speed of 0.63 m/s when maximum belt force and control frequency of the treadmill were low. In addition, the horizontal GRF at a walking speed of 0.63 m/s had more frequent fluctuations than at 1.33 m/s (Figure 10). As the walking speed decreased, the single-leg support time of the stance phase, which is more vulnerable to external force perturbations than the double-leg support phase (Hebenstreit et al., 2015), increased. Increased single-leg support time might result in falls and differences in the joint kinematics.

Tielke reported that belt speed error increased with the body mass and walking speed, based on walking experiments on a treadmill, which could not serve as an inertial frame of reference (Tielke et al., 2019). However, in that study, the effects of treadmill power and control frequency on belt speed error and GRF, and the effects of belt speed error and GRF on body kinematics were not analyzed. In this study, the effects of the maximum belt force and control frequency of the treadmill, body mass, and walking speed on the differences in joint kinematics between overground and treadmill conditions were analyzed.

A motor with high rated power can provide large torque and force (Zhang et al., 2006). This study showed that a treadmill with high motor power and high control frequency reduced the kinematic differences from overground walking. In addition, it was demonstrated that in a specific range of the maximum belt force (exceeding 400 N) and control frequency of the treadmill (exceeding 25 Hz), the RMS differences remained within 3°; however, beyond that range, the RMS differences increased significantly. These results suggest that to reliably achieve conditions during treadmill walking that are most similar to conditions during overground walking, belt forces should be larger than 400 N while the control frequency of the treadmill should be higher than 25 Hz. Not all physical treadmills are ideal. Treadmills are controlled by proportional–integral–derivative controllers in rehabilitation and gait analysis (Su et al., 2007; Von et al., 2007; Weng et al., 2010). Therefore, when designing a treadmill, this study can serve as a guideline to specify the minimum treadmill power and control frequency based on the subject’s body mass and walking speed to reduce kinematic changes as compared with overground walking.

Although we investigated the most influential physical factors of treadmill walking, further physical factors such as the elasticity of the belt and the vibration and damping of the deck may also play a role but were not considered here. The gait controllers were trained to track measured motions while adapting to gravity and the ground reaction forces during overground walking. The individual human response to external force perturbation such as the change of foot braking force during treadmill walking has not been well understood and was therefore not included in the gait controller training used here.

The kinematics of the lower-limb joints were used as a measure to quantify the differences in the human body kinematics between overground and treadmill walking. An alternative metric would be the movement of the body’s center of mass. In this study, among the factors that resulted in differences between treadmill and overground walking, we focused on the physical factors: the subject’s body mass, walking speed, maximum belt force, and control frequency of the treadmill, and presented a guideline for designing a treadmill. Our approach, which considered the isolated effect of each factor, would allow one to understand the underlying physics and physiologies for different gait kinematics during overground and treadmill walking.

## Data Availability

The datasets presented in this article are not readily available because our IRB approval does not include data sharing. Requests to access the datasets should be directed to Seungbum Koo, skoo@kaist.ac.kr.
